# Radiomics-based tumor heterogeneity augments clinicopathological models for predicting recurrence in high-risk clear cell renal cell carcinoma after nephrectomy

**DOI:** 10.1007/s00261-025-05108-2

**Published:** 2025-07-12

**Authors:** Zhan Feng, Piao Yang, Yaoyao Wu, Zhi Li, Zhengyu Hu, Wenting Lan

**Affiliations:** 1 The First Affiliated Hospital, Zhejiang University School of Medicine, Hangzhou, China; 2 Second People’s Hospital of Yuhang District, Hangzhou, China; 3 The First Affiliated Hospital of Ningbo University, Ningbo, China

**Keywords:** Clear cell renal cell carcinoma, Radiomics, Prognosis

## Abstract

**Purpose:**

To investigate the association between CT radiomics-based tumor heterogeneity and recurrence-free survival (RFS) in high-risk clear cell renal cell carcinoma (ccRCC) after nephrectomy, and to determine whether integrating CT radiomics with clinicopathological model enhances recurrence risk prediction for adjuvant treatment decisions.

**Methods:**

This retrospective study included 194 patients with high-risk ccRCC undergoing nephrectomy. A radiomics model based on random survival forest was developed in the training set, using radiomics features extracted from pre-operative corticomedullary phase images. The performance of radiomics, Leibovich score, and the combined model were evaluated using Kaplan-Meier survival analysis, time-dependent receiver operating characteristic curves (time-AUC), time-dependent Brier scores, and decision curve analysis in external test set.

**Results:**

During follow-up, 62 patients experienced recurrence. The radiomics model demonstrated superior predictive performance compared to the Leibovich score, with higher time-dependent AUCs (1-year: 0.882 vs. 0.781; 2-year: 0.865 vs. 0.762; 3-year: 0.793 vs. 0.797; all *p* < 0.05) and better calibration (lower Brier scores) in the test set. Decision curve analysis demonstrated that the combined model provided the highest net benefit, particularly for 2- to 3-year recurrence risk predictions.

**Conclusions:**

For high-risk ccRCC, CT radiomics provides incremental prognostic value beyond conventional clinicopathological models, enabling more precise recurrence risk stratification. This approach bridges imaging and precision oncology, with potential to optimize surveillance protocols and adjuvant therapy trial design.

## Introduction

Clear cell renal cell carcinoma (ccRCC) is one of the most common malignant tumors of the urinary system [1]. For localized or locally advanced non-metastatic renal cell carcinoma (RCC), surgical resection of the tumor or kidney remains the standard treatment [2]. Although the 5-year survival rate for most patients with localized renal cancer can reach 80–95%, the recurrence and metastasis rates for patients with non-metastatic renal cancer who are at high-risk of recurrence are as high as over 30% [3]. Consequently, their overall survival rates and duration of survival are significantly lower than those of patients with localized renal cancer.

Prognostic variability in ccRCC underscores the need for optimized postoperative surveillance, follow-up protocols, and adjuvant therapies. Although adjuvant targeted therapies have shown limited success [4–6], immunotherapy (e.g., KEYNOTE-564 trial) demonstrates promise [7]. Nonetheless, exposing all ccRCC patients to the potential toxic side effects of adjuvant immunotherapy could lead to overtreatment. Currently, there are various clinicopathological models used to predict the postoperative recurrence risk of ccRCC patients, including the University of California, Los Angeles, Integrated Staging System (UISS) [8], Stage, Size, Grade, and Necrosis (SSIGN), and the Leibovich score, among others. The Leibovich score, an improvement based on the SSIGN, is widely used to assess the prognosis after nephrectomy for non-metastatic ccRCC [9]. However, these models fail to account for tumor heterogeneity, a critical factor in tumor evolution [10], limiting their ability to guide personalized therapy for patients at varying recurrence risks.

CT is indispensable in ccRCC diagnosis and management. Radiomics deciphers imaging patterns reflective of tumor biology, quantifying heterogeneity to augment prognostic insights. Prior work predominantly assessed malignancy risk, histology, and tumor grade [11], Only a limited number of investigations have explored the association between radiomics and survival outcomes in ccRCC patients [12–14], and none have specifically addressed high-risk ccRCC.

In this multicenter study, we investigated the association between radiomic features and clinical outcomes in ccRCC patients, evaluating whether integration of radiomic signatures with clinicopathological models could enhance predictive accuracy for recurrence-free survival (RFS) following surgical resection in high-risk ccRCC cohorts, potentially refining adjuvant therapy selection.

## Materials and methods

### Patients

**Fig. 1:**
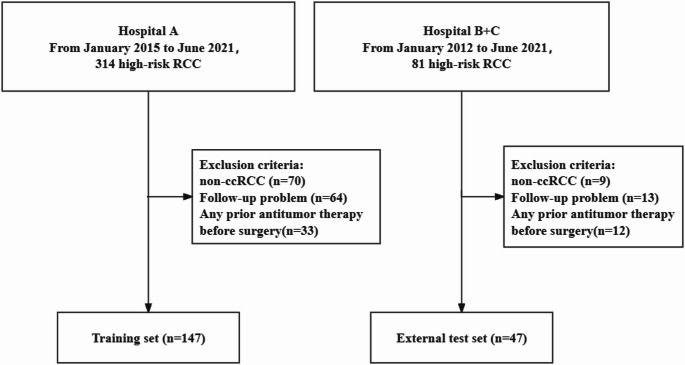
Study population flowchart

We retrospectively analyzed data from three hospitals, including patients with surgically resected, pathologically confirmed RCC, divided into model training and external test cohorts. High-risk non-metastatic ccRCC inclusion criteria (adapted from S-TRAC) comprised: (1) histologically confirmed RCC (T3-T4N0Gany or TanyN1Gany); (2) contrast-CT scan less than 14 days before surgery; (3) Eastern Cooperative Oncology Group Performance Status (ECOG-PS) score ≤ 2 before nephrectomy. The exclusion criteria included: (1) incomplete clinicopathologic and follow-up data; (2) non-clear cell carcinoma; (3) any prior antitumor therapy before surgery. Prior to enrollment, blinded review of CT images was required to confirm the absence of macroscopically visible residual or metastatic disease following nephrectomy. The workflow of the study is illustrated in Fig. 1.

This retrospective observational study adhered to the ethical standards established by the Declaration of Helsinki. The study was approved by the Scientific research IRB of First Affiliated Hospital of Zhejiang University School of Medicine (IIT No.0531), and the requirement for informed consent was waived.

### Clinical data and followup

The following data were collected: age, gender, Fuhrman grade, pathological T and N stage, ECOG-PS. The endpoint of this study was RFS. RFS was calculated from the date of surgery to the earliest occurrence of recurrence, metastasis, or death without recurrence; patients who remained disease-free were censored at the date of their last negative follow-up. All recurrence events were confirmed by imaging or pathological examination when available. Patients were postoperatively followed up quarterly for the first 2 years and then 6 months.

### CT protocols and image segmentation

The CT images were acquired from five different CT scanners. The scanning coverage encompassed both kidneys and any detectable masses. Imaging was performed during the corticomedullary phase (25–28 s) and nephrographic phase (65–70 s) following administration of 80–100 mL of iodinated contrast medium (300 mg/mL) at an injection rate of 2.5–3.0 mL/s. The specific scanning and reconstruction parameters for each of the five CT scanners are detailed in Table 1.

**Table 1:** Scanning parameters for renal CT examination

CT scanner	CT256	CT320	CT64	CT64	CT64
Scanner model	Brilliance-ICT	Revolution EVO	Definition Flash	Aquilion ONE	Revolution Frontier ES
Manufacturer	Philips	General Electric	SIEMENS	Toshiba	General Electric
Tube voltage (Kv)	120	120	120	120	120
Tube current (mAs)	300–350	Automas 300–350	CAREDose 4D 350	AIRDR 3D 350	Automas 200–420
Kernel	Standard(B)	Standard	B30f	Fc10	Standard
Collimation (mm)	128*0.625	64*0.625	64*0.6	160*0.625	64*0.625
Slice thickness (mm)	5	5	5	5	5
Field of view (mm²)	360 × 360	360 × 360	360 × 360	360 × 360	360 × 360
Matrix	512 × 512	512 × 512	512 × 512	512 × 512	512 × 512

**Fig. 2:**
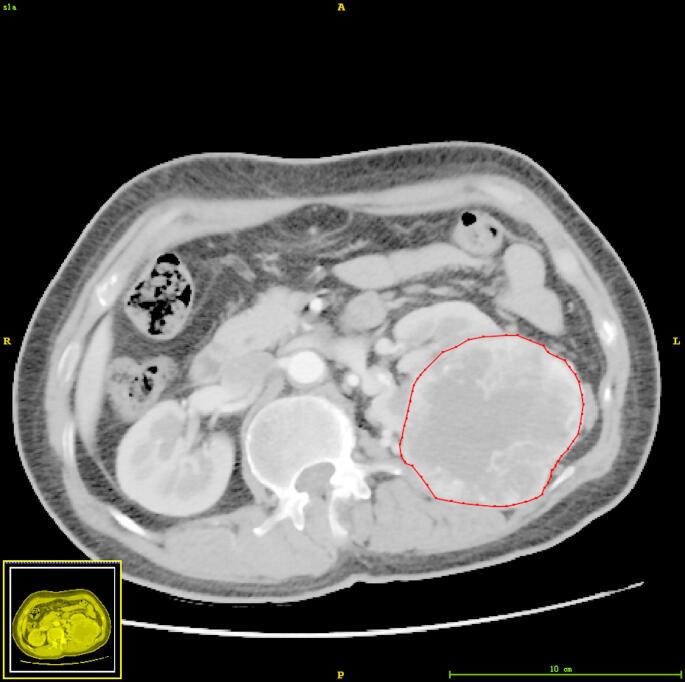
Tumor region of interest delineation diagram

Tumors were manually delineated on all tumor containing axial slices on corticomedullary phase using ITK-Snap software (Fig. 2). The region of interest was limited to the primary tumor, with exclusion of lymph nodes and vascular structures such as tumor thrombus. Segmentations were initially performed by a radiologist (YYW, with 3 years of experience), who remained blinded to patient information. All segmentation contours were reviewed and, if necessary, revised by a senior radiologist (ZF, with over 20 years of experience). To assess intra-rater reproducibility, another radiologist (PY, with 8 years of experience) independently delineated 30 randomly selected cases. Intraclass correlation coefficient (ICC) greater than 0.75 was considered satisfactory reliability and reproducibility.

### Radiomics model development

The radiomics model development workflow encompasses three main steps: feature extraction, feature selection, and model construction.

Before radiomics feature extraction, all images normalized to a grayscale level of 1–64 and the voxel was resampled to 1 × 1 × 1 mm^3^ using linear interpolation. A total of 107 radiomics features —encompassing intensity statistics, shape, and texture features—were extracted using Pyradiomics (version 3.0) [15], which is compliant with the Image Biomarker Standardization Initiative (IBSI) [16].

Feature dimension reduction was conducted on the training dataset. First, the features with ICCs ≤ 0.75 were removed. Second, the Spearman rank correlation test was used to investigate the internal linear correlation between individual features. Third, the Boruta algorithm selects the most important variables relevant to clinical outcomes. Following feature filtering, a Random Survival Forest (RSF) model was constructed to predict RFS. The optimal model parameters were selected using 10-fold cross-validation on the training set. The importance of radiomics features was analyzed and visualized using Shapley additive explanation analysis.

### Models validation

The Leibovich score was calculated as follows: 3 (pT2 tumors) + 4 (pT3 tumors) + 4 (pT4 tumors) + 2 (pN1 or pN2) + 1 (tumor size ≥ 10 cm) + 1 (nuclear grade 3) + 3 (nuclear grade 4) + 1 (histologic tumor necrosis), with 0 points assigned for absent features. Patients were categorized into low risk (0–2 points), intermediate risk (3–5 points), and high risk (≥ 6 points) groups based on their total scores.

A radiomics-Leibovich combined model was developed by integrating the Leibovich score as a feature with radiomic features to construct a fused predictive model. All models were validated on an external test cohort. The discriminative ability of the models was evaluated using receiver operating characteristic (ROC) curves, while the accuracy of probability predictions was assessed using the Brier score. The clinical utility of the models in potential treatment decision-making was evaluated through decision curve analysis (DCA) [17]. The radiomics and combined models were stratified using the median risk score derived from the training cohort, while the Leibovich score retained the previously established cutoff value of 6 for grouping. Kaplan-Meier analysis was employed to estimate recurrence-free survival (RFS) differences between groups, and the log-rank test was used for statistical comparison.

### Statistical analysis

Continuous variables are presented as mean ± standard deviation (SD) or median (interquartile range [IQR]). Categorical variables were reported as counts (*n*) and percentages (%). When comparing normally distributed continuous variables, the independent samples T-test was employed. For non-normally distributed continuous variables, the Mann-Whitney U test was utilized. The chi-square test was implemented to analyze differences in the distribution of categorical variables. The area under the curve (AUC) values for 1-year, 2-year, and 3-year predictions were compared using DeLong’s test.

Statistical analyses were performed with R statistical software (version 4.4.2, R Foundation for Statistical Computing). All statistical tests were two-sided, and *p* value less than 0.05 was considered to indicate statistical significance.

## Results

### Patient characteristics

The clinical characteristics are shown in Table 2. The median duration of follow-up was 56 months (95% confidence interval [CI], 51 to 60). During follow-up, 62 patients experienced recurrence, post-operative RFS rate for the entire cohort was 68%.

**Table 2:** Clinical characteristics of the high-risk CcRCC patients

Characteristic	Total *N* = 194	Training Set *N* = 147	Test Set *N* = 47	*p* value
Age, Mean ± SD, (years)	59 ± 10	59 ± 11	58 ± 10	0.810
Gender, *n* (%)				0.737
Female	42 (21.6%)	31 (21.1%)	11 (23.4%)	
Male	152 (78.4%)	116 (78.9%)	36 (76.6%)	
Fuhrman grade, *n* (%)				0.614
2	4 (2.1%)	4 (2.7%)	0 (0.0%)	
3	178 (91.8%)	133 (90.5%)	45 (95.7%)	
4	12 (6.2%)	10 (6.8%)	2 (4.3%)	
Location, *n* (%)				0.795
Left	100 (51.5%)	75 (51.0%)	25 (53.2%)	
Right	94 (48.5%)	72 (49.0%)	22 (46.8%)	
Pathological T stage, *n* (%)				0.117
T3	185 (95.4%)	138 (93.9%)	47 (100.0%)	
T4	9 (4.6%)	9 (6.1%)	0 (0.0%)	
Lymph node metastasis, *n* (%)				0.106
No	134 (69.1%)	106 (72.1%)	28 (59.6%)	
Yes	60 (30.9%)	41 (27.9%)	19 (40.4%)	
ECOG-PS, *n* (%)				0.099
0	167 (86.1%)	128 (87.1%)	39 (83.0%)	
1	25 (12.9%)	19 (12.9%)	6 (12.8%)	
2	2 (1.0%)	0 (0.0%)	2 (4.3%)	
Leibovich score, *n* (%)				0.725
High	62 (32.0%)	46 (31.3%)	16 (34.0%)	
Intermediate	132 (68.0%)	101 (68.7%)	31 (66.0%)	

ccRCC clear cell renal cell carcinoma; ECOG-PS Eastern Cooperative Oncology Group Performance Status

### Radiomics model development

**Fig. 3:**
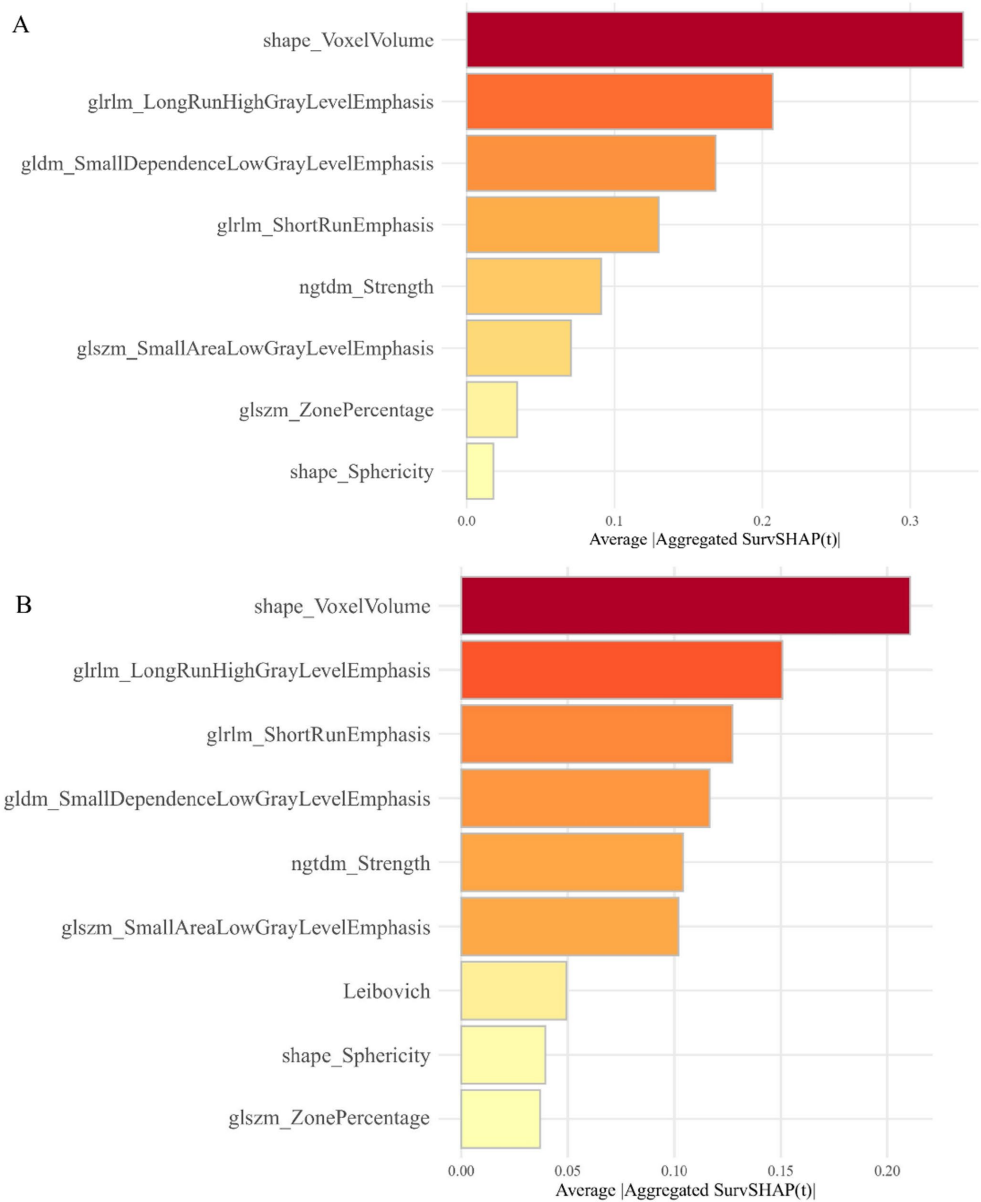
The bar chart illustrates the impact of each feature on the Random Survival Forest model (A) and combined model (B) based on Shapley Additive explanation. glrlm: gray-level run-length matrix; gldm: gray-level dependence matrix; ngtdm: neighboring gray-tone difference matrix; glszm: gray-level size zone matrix

45 radiomics features demonstrated robust inter-observer consistency. Following Spearman correlation testing and Boruta feature selection, eight radiomics features associated with RFS were identified and subsequently incorporated into the RSF model (Fig. 3).

### Model validation

**Fig. 4:**
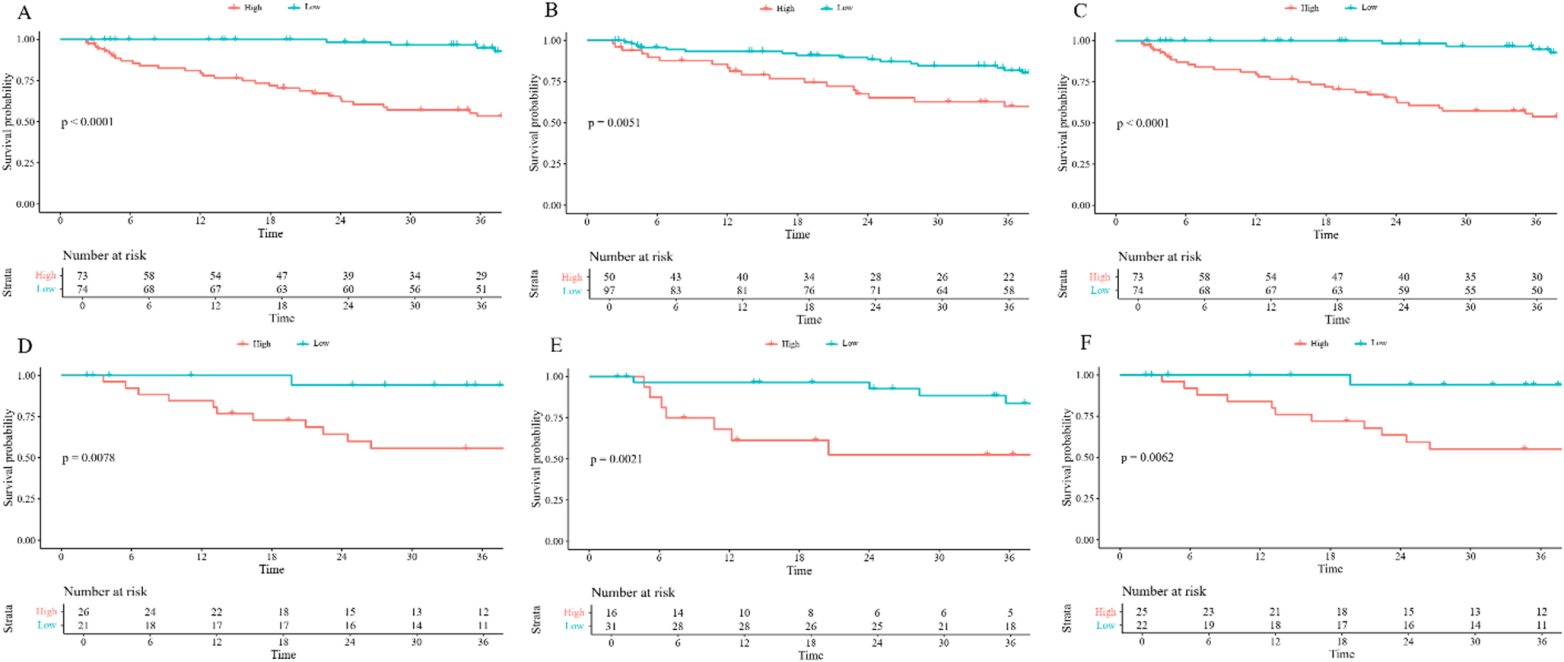
Kaplan-Meier survival curves for recurrence-free survival (RFS) by radiomics, Leibovich score and combined model. (A, D) Kaplan-Meier survival curves based on the radiomics in the training (A) and test (D) sets. Patients were classified into high- and low-risk groups using the median risk score derived from the model in the training set. (B, E) The curves based on the Leibovich score in the training (B) and test (E) sets. A cutoff of ≥ 6 was used to define the high-risk group. (C, F) The curves based on the combined model in the training (C) and test (F) sets. The same median value from the combined model was used for risk stratification

As shown in Table 3, in the training set, the time-dependent AUC of the radiomics model for predicting RFS were as follows: with 1-year AUC of 0.929 (95% CI: 0.873–0.986), 2-year AUC of 0.895 (95% CI: 0.834–0.956), and 3-year AUC of 0.886 (95% CI: 0.824–0.949). However, the AUC values in the test set showed a decline, with 1-year AUC of 0.882 (95% CI: 0.779–0.986), 2-year AUC of 0.865 (95% CI: 0.707–1), and 3-year AUC of 0.793 (95% CI: 0.616–0.969). Kaplan-Meier analysis revealed a significant difference in RFS between the stratified high- and low-risk groups (log-rank *p* < 0.05; Fig. 4).

**Table 3:** The performance of models in the test set

	Radiomics	Leibovich score	Combined
1-year AUC (95% CI)	0.882 (0.779,0.986)	0.781 (0.586,0.976)	0.853 (0.726,0.980)
2-year AUC (95% CI)	0.865 (0.707,1)	0.762 (0.612,0.912)	0.851 (0.687,1)
3-year AUC (95% CI)	0.793 (0.616,0.969)	0.718 (0.658,0.937)	0.780 (0.588,0.973)
1-year Brier score (95% CI)	0.086 (0.027,0.144)	0.087 (0.026,0.147)	0.084 (0.024, 0.144)
2-year Brier score (95% CI)	0.126 (0.069,0.182)	0.156 (0.089,0.222)	0.129 (0.071,0.187)
3-year Brier score (95% CI)	0.149 (0.090,0.207)	0.161 (0.103,0.218)	0.150 (0.090,0.209)

AUC area under the curve; CI confidence interval

In the test set. The AUC values of the Leibovich score for predicting RFS were as follows: with 1-year AUC of 0.781 (95% CI: 0.586–0.976), 2-year AUC of 0.762 (95% CI: 0.612–0.912), and 3-year AUC of 0.797 (95% CI: 0.658–0.937). The combined model achieved superior discrimination, with AUCs of 0.853 (95% CI: 0.726–0.980), 0.851 (95% CI: 0.687-1), and 0.780 (95% CI: 0.588–0.973) for 1-, 2-, and 3-year RFS prediction, respectively. Kaplan-Meier analysis confirmed significant stratification of recurrence risk by both the Leibovich score and combined model (Fig. 4).

**Fig. 5:**
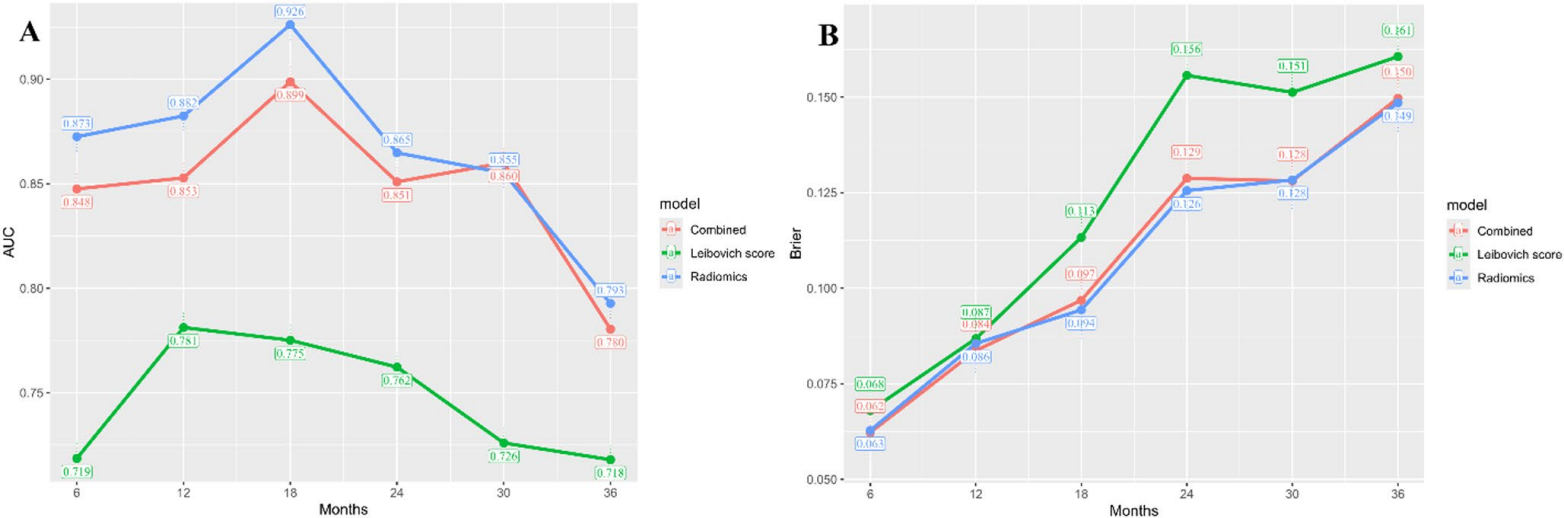
The time-dependent area under the curve (A) and Brier score (B) in test set

**Fig. 6:**
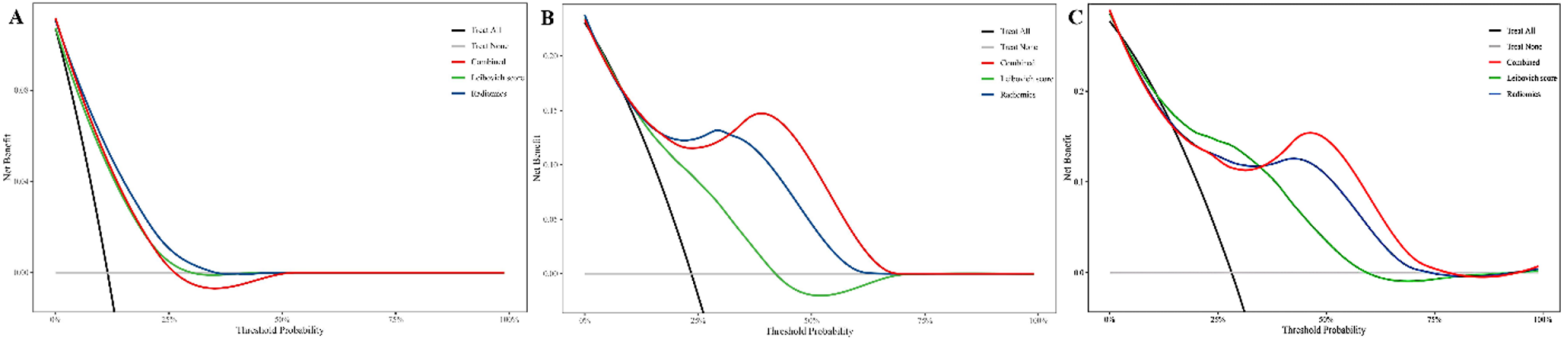
Decision curve analysis of models in the prediction of 1- (A), 2- (B), and 3- (C) year recurrence-free survival in patients with high-risk clear cell renal cell carcinoma

DeLong’s test revealed that both the radiomics model and combined model showed significantly higher AUCs than the Leibovich score at 1-, 2-, and 3-year timepoints (all *p* < 0.05), while no significant difference was observed between the radiomics and combined models (Fig. 5).The Brier score, used to evaluate model accuracy and as a quantitative metric for calibration curves, indicates higher accuracy with lower values. The time-dependent Brier scores demonstrates that the accuracy of the radiomics model and combined model consistently surpasses that of the Leibovich score(Fig. 5). As shown in Fig. 6, all models provided net benefits in adjuvant treatment decision-making compared to the default strategies (“treat all” or “treat none”). Notably, combined model demonstrated significantly higher overall net benefits at 2-, and 3-year for RFS prediction compared to radiomics the Leibovich score.

## Discussion

This multicenter study demonstrates that radiomic analysis significantly predicts RFS in high-risk ccRCC patients following nephrectomy. Our radiomics model showed superior predictive performance compared to the Leibovich score. Integrating radiomic features with prognostic models enhanced RFS prediction accuracy. When applied to guide adjuvant treatment decisions for high-risk ccRCC patients, the combined model achieved relatively higher net benefits.

Various clinicopathological prognostic models have been developed to predict survival outcomes in ccRCC. Previous studies have shown that the Leibovich score outperforms the UISS in predicting all survival indicators [18, 19] and has been applied in the SORCE clinical trial [20]. Our results indicate that the Leibovich score exhibited stable predictive performance over the 3-year study period, with AUC values consistently around 0.70. As Correa et al. demonstrated in their evaluation of nine RCC prognostic models, the predictive ability of such models tends to peak within the first two years and gradually declines thereafter, as late recurrence becomes increasingly influenced by a complex interplay between the patients’ clinical status and tumor biology [18]. Therefore, based on our own data and study design, we selected 3-year RFS as the primary endpoint.

While traditional tumor staging and pathological grading systems provide fundamental stratification for clinical practice, they are insufficient to fully meet the demands of individualized treatment in precision medicine [21]. This study confirms radiomics as an effective approach for tumor evaluation, showing its association with postoperative RFS in high-risk ccRCC. Among numerous radiomics parameters (Fig. 2), tumor volume contributed most to the model, consistent with the traditional view that preoperative tumor size as a predictor of disease recurrence has been widely validated in multiple studies [22–24].

Intratumoral heterogeneity plays a critical role in tumor progression and is closely linked to poor clinical outcomes [10]. Radiomics offers a non-invasive means to quantify this heterogeneity through advanced image features, providing insights into tumor biology that go beyond what conventional imaging or histopathology can reveal. Features such as those derived from the gray-level run length matrix and gray-level dependence matrix are particularly effective in capturing spatial variations in tumor intensity patterns. These mathematical models describe how pixel intensities are distributed and how they relate to neighboring pixels, thereby quantifying tumor texture and structural complexity. As a result, texture features reflect underlying tissue characteristics associated with hypoxia, necrosis, and angiogenesis [25, 26], enabling radiomics to better characterize the tumor microenvironment and improve the prediction of ccRCC prognosis [27].

We compared the performance of the radiomics model and the Leibovich score in predicting 1-, 2-, and 3-year RFS. The radiomics model consistently demonstrated superior discrimination, calibration, and clinical utility across all time points, significantly outperforming the Leibovich score. These findings are consistent with prior imaging studies [10, 25], which suggest that radiomics features capture additional tumor heterogeneity information beyond conventional clinicopathological variables. Multiple recent studies have shown that quantitative radiomics features can be effectively integrated into prognostic modeling frameworks [28, 29]. Our study found that although a combined radiomics-clinical model showed modest improvement over the clinical model alone (ΔAUC 0.07–0.09), its accuracy closely approximated that of the radiomics-only model. However, DCA suggested a slight clinical advantage of the combined model. We speculate that there is substantial overlap in the prognostic information captured by variables in the two models, particularly with regard to tumor size. Several studies have also suggested a strong association between CT-derived texture features and key histopathological characteristics, such as tumor grade and nuclear morphology [30]. This may explain why the addition of the Leibovich score resulted in only marginal improvements in model performance.

Furthermore, our study cohort differs markedly from prior radiological investigations. Notably, among numerous adjuvant therapy trials for high-risk RCC, most have yielded negative results, which may be attributed in part to the high heterogeneity in inclusion criteria. Some studies used the Leibovich score to define high risk, such as the SORCE trial, while others employed TNM staging, often with inconsistent definitions of T-stage. Based on this, our study collected a homogeneous cohort of high-risk non-metastatic ccRCC patients, whereas previous ccRCC survival prognostic studies have shown significant heterogeneity in cohort design [13, 30]. Many ccRCC postoperative recurrence imaging studies included a high proportion of T1 stage patients, sometimes exceeding 70% [28]. In fact, for T1N0M0 localized renal cancer, where the tumor has not breached the renal capsule, surgical treatment typically yields good outcomes, with 5-year overall survival rates reaching 80-95%. Heterogeneous cohorts dominated by T1 stage tumors undoubtedly limit model generalizability and utility in adjuvant treatment decision-making.

Our study has several limitations. First, the relatively small sample size introduces unavoidable selection bias and sample imbalance, necessitating well-designed prospective radiomics studies for robust validation and the establishment of risk thresholds. Second, the radiomics parameters were extracted exclusively from the corticomedullary phase, without incorporating other phases. Previous research suggests that radiomics models based on multiphase CT imaging may outperform those derived from single-phase imaging [31]. Third, we performed a Radiomics Quality Score self-assessment [32], which yielded a total score of 22. This assessment indicates acceptable methodological rigor; however, areas such as cost-effectiveness analysis and exploration of biological correlates require further improvement in future studies. Fourth, our survival analysis included only RFS as the primary endpoint. The inclusion of additional endpoints such as cancer-specific survival and overall survival would provide a more comprehensive evaluation models prognostic performance. Finally, we did not account for potential clinical complications following nephrectomy, such as acute or chronic kidney injury. These postoperative conditions may limit the feasibility of administering adjuvant treatments and could therefore indirectly influence survival outcomes. Future studies should consider these factors to improve the accuracy of risk stratification and treatment planning.

## Conclusion

For high-risk ccRCC, accurate postoperative prognostic assessment forms the foundation for formulating rational clinical decisions. The radiomics-based tumor heterogeneity can predict RFS and add incremental prognostic value to the Leibovich score models. It holds the potential to bridge the gap between imaging and precision medicine, optimize patient monitoring strategies, and improve the design of adjuvant therapy clinical trials.

## Data Availability

No datasets were generated or analysed during the current study.

## Abbreviations

ccRCC: Clear cell renal cell carcinoma
RCC: Renal cell carcinoma
UISS: University of California, Los Angeles, Integrated Staging System
SSIGN: Stage, size, grade, and necrosis
ECOG-PS: Eastern Cooperative Oncology Group Performance Status
RFS: Recurrence-free survival
CI: Confidence interval
RSF: Random survival forest
AUC: Area under the curve
ROC: Receiver operating characteristic
ICC: Intra-class correlation coefficient
IBSI: Image Biomarker Standardization Initiative
DCA: Decision curve analysis
